# Upregulation of the Wnt Co-Receptor LRP6 Promotes Hepatocarcinogenesis and Enhances Cell Invasion

**DOI:** 10.1371/journal.pone.0036565

**Published:** 2012-05-03

**Authors:** Edmund Kwok-Kwan Tung, Betty Yin-Chi Wong, Tai-On Yau, Irene Oi-Lin Ng

**Affiliations:** 1 State Key Laboratory for Liver Research, The University of Hong Kong, Pokfulam, Hong Kong; 2 Department of Pathology, The University of Hong Kong, Pokfulam, Hong Kong; University of Nebraska Medical Center, United States of America

## Abstract

**Background:**

Activation of the Wnt/β-catenin signaling pathway plays a crucial role in hepatocellular carcinoma (HCC). Low-density lipoprotein (LDL) receptor-related protein-6 (LRP6) is one of the co-receptors of the Wnt/β-catenin pathway and forms a signaling complex with Wnt ligand and Frizzled receptor to activate downstream signaling. However, the role of LRP6 in hepatocarcinogenesis is unclear. In this study, we examined its expression and roles in human HCC.

**Methodology/Principal Findings:**

Using real-time quantitative RT-PCR, we found that LRP6 was frequently (45%) overexpressed in human HCCs (P = 0.003). *In vitro* studies showed that ectopic expression of LRP6 increased the protein level of β-catenin. Moreover, overexpression of the full-length and constitutively active LRP6, respectively, activated the WNT/β-catenin signaling pathway, as shown by the TCF/β-catenin reporter assay. With regard to the effects of LRP6 overexpression in HCC cells, stable overexpression of the constitutively active LRP6 in BEL-7402 HCC cells enhanced cell proliferation, cell migration, and invasion *in vitro* as well as tumorigenicity in nude mice.

**Conclusions/Significance:**

Our findings indicate that overexpression of LRP6 contributes to the hyperactivation of the Wnt/β-catenin signaling pathway in human HCCs and suggest it may play a role in hepatocarcinogenesis.

## Introduction

Hepatocellular carcinoma (HCC) is the sixth most common cancer worldwide and particularly prevalent in Eastern and Southeast Asia and Africa [1]. Aberrant activation of Wnt/β-catenin signaling pathway is closely associated with the formation of HCC [2], [3], [4], [5], [6], [7], [8]. Without Wnt ligands, β-catenin is constantly degraded by a destruction complex containing a scaffold protein Axin and two kinases GSK3β and CK1. Mechanistically, β-catenin is phosphorylated by GSK3β and then ubiquitinated by E3 ligase β-Trcp for degradation. Upon Wnt activation, β-catenin is no longer targeted for degradation but accumulates in the cytoplasm and translocates into the nucleus. Nuclear β-catenin binds TCF transcription factors to initiate the expression of Wnt target genes, like c-myc and cyclin D1, to promote cell growth and development. It has been demonstrated that hyperactivation of this pathway plays an important role in carcinogenesis and other diseases (for review, see [9], [10]). Accumulation of β-catenin in the cytoplasm and nucleus is the hallmark of the Wnt-driven carcinogenesis. In our previous study, we reported that β-catenin gene mutations at exon 3 were found in about 12% in human HCCs and the mutations at this site contributed to accumulation of β-catenin in HCC [11]. Interestingly, accumulation of the β-catenin in the cytoplasm was about 62% in HCCs. This suggests that the mechanisms leading to β-catenin overexpression may be heterogeneous. We hypothesized that, in addition to β-catenin mutations, hyperactivation of Wnt/β-catenin pathway may be contributed by other alterations in the pathway.

Low-density lipoprotein (LDL) receptor-related protein-6 (LRP6) is one of the co-receptors of Wnt/β-catenin pathway which form a signaling complex with Wnt ligand and Frizzled receptor to activate downstream signaling. Developmental studies have shown that co-receptors LRP5/6 are essential for the activation of Wnt/β-catenin pathway. However, evidence about the role of LRP6 in tumorigenesis is scanty, with only few reports showing the potential oncogenic role of LRP6 in cancers. Li et al. demonstrated that stable expression of LRP6 in human fibrosarcoma HT1080 cells increased the cytosolic β-catenin level and enhanced cell proliferation [12]. Two very recent reports by Bu's group showed that LRP6 was overexpressed in a subpopulation of human breast cancers [13] and, more importantly, demonstrated that activation of the Wnt signaling by overexpressing LRP6 alone was enough to induce breast cancer formation [14]. These studies highlighted the importance and possible role of the cell-surface co-receptor LRP6 level in Wnt-driven carcinogenesis. This may also explain the discrepancy between the frequency of β-catenin mutations and the proportion of β-catenin accumulation in human HCCs. Based on this rationale, we hypothesized that overexpression of the cell surface LRPs contributed to the accumulation of β-catenin and Wnt-driven hepatocarcinogenesis. This study investigated the expression pattern of LRP6 and its possible role in human HCCs.

Our findings showed that LRP6 was significantly up-regulated in human HCCs. Ectopic expression of constitutively active LRP6 activated Wnt/β-catenin signaling in HCC cells and promoted cell proliferation, migration and invasion *in vitro*. LRP6 also enhanced tumor growth *in vivo*. Our findings indicate that overexpression of LRP6 contributes to the hyperactivation of Wnt/β-catenin signaling pathway and suggest it plays a role in hepatocarcinogenesis.

## Results

### Overexpression of LRP6 in human HCCs

**Figure 1:**
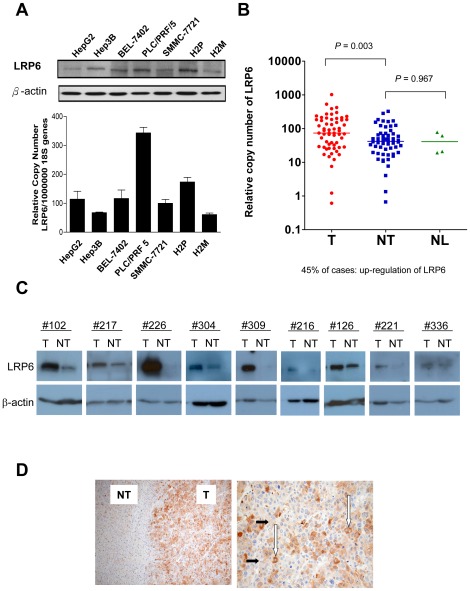
Overexpression of LRP6 in HCC cell lines and human HCCs. (A) Quantitative real-time PCR and Western blotting. Both transcripts and protein of LRP6 were expressed in all seven HCC cell lines. (B) In human HCCs, the transcript level of LRP6 was frequently (45%) up-regulated as compared with their corresponding non-tumorous livers (P = 0.003). (C) Western blot analysis. LRP6 protein level was determined in 28 HCC pairs and was found to be overexpressed in 9 (32%) cases. The 9 cases with LRP6 protein overexpression in the tumors are shown. (D) Immunohistochemical analysis. LRP6 protein was found to be overexpressed in the HCC as compared with the corresponding non-tumorous liver. High power magnification of the tumor showed strong positive staining of LRP6 protein in the cytoplasm (white arrows) and also the membranes (black short arrows) of the tumor cells.

The expression pattern of LRP6 has not been reported in human HCCs. Therefore, we first examined the expression pattern of LRP6, at both transcript and protein levels, in human HCC cell lines. Both transcripts and protein of LRP6 were expressed in all seven HCC cell lines tested (Figure 1A). Then, we investigated its transcript levels in 60 pairs of human HCCs and their corresponding non-tumorous livers. The transcript level of LRP6 was frequently (45%) and significantly up-regulated in human HCCs, as compared with their corresponding non-tumorous livers (P = 0.003, Mann Whitney test) and normal liver tissues (P = 0.003) (Figure 1B). Cases with T/NT ratio more than 2 folds were defined as overexpression and those with ratio less than 0.5 as underexpression. Twenty-seven (45%) of the 60 cases were found to have LRP6 overexpression and the range of the T/NT ratio was from 2.07 to 23.7 folds. In contrast, only 2 cases had T/NT ratio below 0.5, and 12 cases between 0.5–1.0. We next performed Western blotting to evaluate the protein levels of LRP6 on 28 pairs of HCCs, randomly selected from these 60 pairs with mRNA levels examined. Overexpression of LRP6 at protein level was seen in 9 cases (32%) (Figure 1C). Four of these 9 cases had upregulation of both LRP6 transcript and protein levels. With immunohistochemistry, strong positive staining of LRP6 was seen in the cytoplasm (white arrows) and cell membranes (black short arrows) of the tumor cells (Figure 1D). Clinicopathological analysis, however, showed no significant correlation of LRP6 overexpression at mRNA level with any of the clinicopathological features (Table 1). To examine the relationship between LRP6 and β-catenin expression, we compared the LRP6 transcript level with β-catenin using immunohistochemical staining in 36 human HCC cases. Although 8 (22%) of the cases showed upregulation of both LRP6 transcript and β-catenin protein levels, there was no significant association between LRP6 overexpression and β-catenin overexpression (Table 1).

**Table 1:** Clinicopathologic correlation of LRP6 transcript in HCC patients.

Parameters		No LRP6 overexpression	LRP6 overexpression (≥2-fold)	P-value
Gender	Male	23	20	0.767
	Female	9	6	
Age	<60	21	13	0.288
	≥60	11	13	
Tumor stage (TNM)	Early (I & II)	12	14	0.290
	Advanced (III & IV)	20	12	
Tumor size (cm)	<5 cm	8	10	0.388
	≥5 cm	23	15	
Cellular differentiation (Edmondson grading)	Better differentiation (grades I & II)	14	13	0.590
	Poorer differentiation (grades III & IV)	18	11	
Tumor encapsulation	Absent	21	13	0.278
	Present	10	12	
Tumor microsatellite	Absent	18	13	1.000
	Present	14	11	
Venous permeation	Absent	15	14	0.792
	Present	17	12	
Non-tumorous liver	Non-cirrhotic	21	12	0.280
	Cirrhotic	11	13	
Serum HBsAg	Positive	29	19	0.161
	Negative	3	6	
β-catenin overexpression	Absent	8	11	0.739
By IHC staining*	Present	9	8	

IHC, immunohistochemical;

* Nuclear stain or cytoplasmic overexpression.

### Transient expression of either full length or constitutively active form of LRP6 activated the Wnt/β-catenin pathway

**Figure 2:**
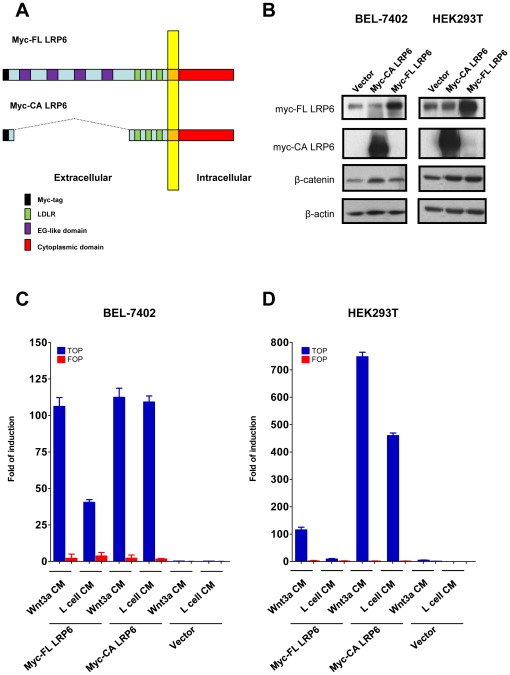
Ectopic expression of LRP6 activated the Wnt/β-catenin pathway. (A) Schematic diagram showing the structural domains of Myc-tagged full length form of LRP6 (myc-FL LRP6) and the constitutively active form (myc-CA LRP6). (B) Western blotting. Myc-FL LRP6 and myc-CA LRP6 were transiently overexpressed in BEL-7402 HCC cell line and human embryonic kidney cell HEK293T cells. The protein level of β-catenin was increased in both BEL-7402 HCC cell line and HEK293T cells. (C) TOP/FOP luciferase reporter assay. Expression of myc-FL LRP6 led to an activation of TCF/β-catenin reporter up to ∼40-fold (without Wnt3a treatment) and ∼120-fold (with Wnt3a treatment), respectively, as compared with the vector control. The fold of induction in myc-CA LRP6 cells reached ∼150-fold even without Wnt3a treatment. (D) Similar results were also observed in HEK293T cells.

To investigate the mechanistic role of LRP6 overexpression on Wnt/β-catenin pathway, we performed *in vitro* studies to see whether Wnt/β-catenin pathway was activated upon ectopic expression of the LRP6 constructs. Myc-tagged full-length form of LRP6 (myc-FL LRP6) and its constitutively active form (myc-CA LRP6) were transiently overexpressed in BEL-7402 HCC cells and HEK293T cells. The myc-CA LRP6 was a truncated form of LRP6 protein lacking 4 EGF-like repeats (Figure 2A). It was found to be a constitutively activated form of LRP6 as demonstrated by Brown's group [15]. From the result, the protein levels of β-catenin were increased upon ectopic expression of either myc-FL LRP6 or myc-CA LRP6 in both BEL-7402 HCC cell line and HEK293T cells (Figures 2B).

To investigate the effect of LRP6 overexpression on the transcriptional activity of Wnt/β-catenin pathway, TCF/β-catenin reporter assay (TOP/FOP luciferase reporter assay) was done on both BEL-7402 and HEK293T cells. Our data showed that overexpression of either myc-FL LRP6 or myc-CA LRP6 led to an activation of TCF/β-catenin reporter up to ∼40 and ∼110 folds, respectively, as compared with the vector control (Figure 2C). Upon Wnt3a treatment, the induction of luciferase signal in myc-FL LRP6-transfected cells reached ∼112-fold, similar to the level of the cells transfected with the constitutively active form (myc-CA LRP6) with or without Wnt3a treatment. Similar results were also observed in HEK293T cells (Figure 2D).

### Stable expression of constitutively active form of LRP6 activated Wnt/β-catenin signaling in HCC cells

**Figure 3:**
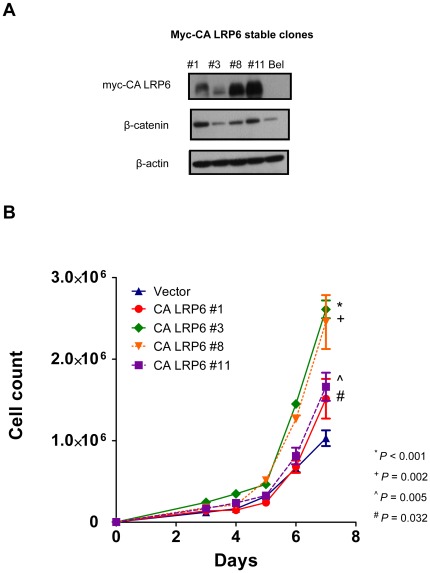
Constitutively active LRP6 enhanced cell proliferation *in vitro.* (A) LRP6 expressing BEL-7402 stable cells were established using myc-CA LRP6 construct. The protein level of β-catenin was upregulated as compared with the parental BEL-7402 cells. (B) Cell proliferation assay. The numbers of cells of CA LRP6 Clones #1, #3, #8 and #11 on Day 7 were significantly higher than the vector control BEL-7402 cells (P = 0.032, <0.001,  = 0.002 and 0.005, respectively).

BEL-7402 cells stably expressing LRP6 were established using the myc-CA LRP6 construct. After the selection process, four stable clones of CA LRP6 cells were established. In these myc-CA LRP6 expressing cells, the protein level of β-catenin was increased as compared with the parental BEL-7402 cells (Figure 3A). It indicates that the Wnt/β-catenin signaling pathway was activated in myc-CA LRP6 expressing BEL-7402 cells.

### LRP6 enhanced cell proliferation *in vitro*

To investigate the role of constitutively active form of LRP6 on cancer cell growth, the four myc-CA LRP6 stably expressing BEL-7402 clones and vector control BEL-7402 cells were seeded onto 6-well plates for cell counting. We found that myc-CA LRP6 enhanced the cell proliferation rate of BEL-7402 cells. The cell numbers on Day 7 in Clones #1, #3, #8, and #11 were significantly higher than that of the vector control BEL-7402 cells (P = 0.032, <0.001,  = 0.002, and 0.005, respectively, Student's t-test) (Figure 3B). The doubling time of each clone were also calculated. The doubling time of vector control, CA LRP6 Clones #1, #3, #8 and #11 were 1.25, 0.85, 0.99, 0.94 and 0.97 days, respectively. These data demonstrated that LRP6 expressing cells had higher proliferative rates than that of the vector control.

### LRP6 enhanced both cell migration and invasion *in vitro*

**Figure 4:**
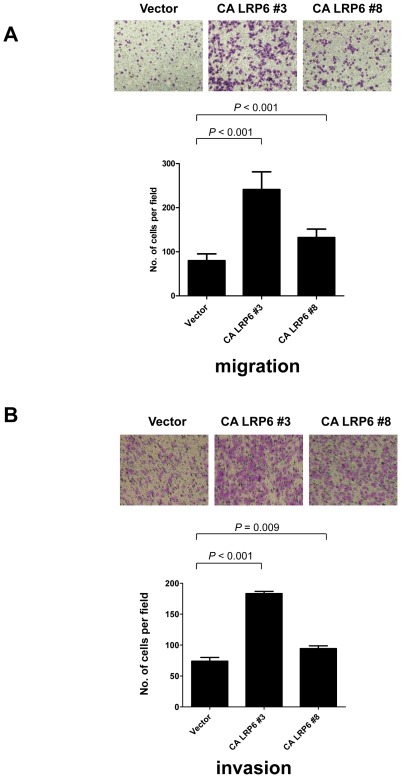
Constitutively active LRP6 promoted both cell migration and invasion. Cell migration and invasion assays were performed using LRP6-stably expressing BEL-7402 cells. (A) Overexpression of myc-CA LRP6 enhanced cell migration in BEL-7402 cells. The numbers of migrated cells in CA LRP6 Clones #3 and #8 were significantly higher than the vector control (P<0.001 for both). (B) Overexpression of myc-CA LRP6 promoted cell invasion in BEL-7402 cells. The numbers of invaded cells in CA LRP6 Clones #3 and #8 were significantly higher than the vector control cells (P<0.001 and  = 0.009, respectively).

Cell migration and invasion assays were performed to test whether the constitutively active form of LRP6 promoted cell migration and invasion in BEL-7402 HCC cells. Overexpression of myc-CA LRP6 enhanced cell motility (Figure 4A). The migrated cells in Clones #3 and #8 were significantly higher in number than the vector control (P<0.001 for both, Student's t-test). In addition, overexpression of myc-CA LRP6 promoted cell invasion in BEL-7402 cells. The numbers of invaded cells in Clones #3 and #8 were significantly higher than that of the vector control BEL-7402 (P<0.001 and  = 0.009, respectively, Student's t-test) (Figure 4B). Similar result was observed in the other two stable clones, Clones #1 and #11 (Figure S1).

### LRP6 enhanced tumor formation *in vivo*

**Figure 5:**
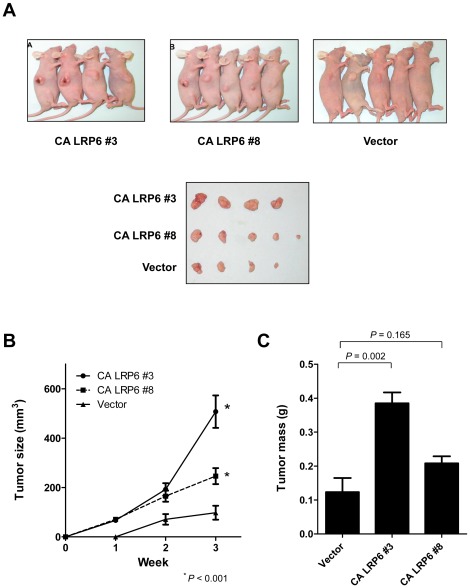
Constitutively active LRP6 enhanced tumor cell growth *in vivo*. (A) *In vivo* nude mice injection assay was performed by injecting myc-CA LRP6 stably expressing and vector control BEL-7402 cells subcutaneously into the flank of the nude mice. (B) The tumor sizes of two myc-CA LRP6 stably expressing tumors were significantly higher as compared with the tumor of vector control (P<0.001). (C) The tumor weight of Clone #3 was higher in myc-CA LRP6 than the control vector (P = 0.002). Another clone (Clone #8) showed a trend of higher tumor weight but the difference did not reach statistical significance (P = 0.165). Error bar = SEM.

Our *in vitro* study demonstrated that myc-CA LRP6 enhanced cell proliferation of BEL-7402 cells. Here, we investigated its role on tumorigenicity and tumor growth in nude mice. Two clones of myc-CA LRP6 and the vector control were injected subcutaneously into the flank of the nude mice. The tumors of the two myc-CA LRP6 stably-expressing tumors were significantly larger than those of the vector control tumors (Figures 5A and 5B) (P<0.001, Student's t-test). In addition, the tumor weight of Clone #3 was significantly higher as compared with the control vector (P = 0.002, Student's t-test). Another clone (Clone #8) also showed a trend of higher tumor weight, although the difference did not reach statistical significance. Similar results were observed in the other two stable clones, Clones #1 and #11 (Figure S2).

## Discussion

Activation of Wnt/β-catenin signaling pathway is well documented to be closely associated with carcinogenesis in different cancers [9], [10], [18]. In human HCC, mutations of β-catenin, APC and Axin genes have been found to contribute the activation of Wnt/β-catenin signaling pathway [19], [20], [21], [22], [23]. However, by frequencies, mutations of the Wnt pathway-related genes in Wnt/β-catenin signaling alone do not entirely explain the dysregulation of the pathway. In this cohort of our HCC patients, mutations of β-catenin at exon 3 was only 11%, while more than 50% of cases showed hyperactivation β-catenin signaling as reflected by β-catenin overexpression in immunohistochemical analysis [11]. β-catenin mutations are not the main cause of dysregulation of Wnt/β-catenin signaling in our HBV-associated HCCs. Our result is consistent with that of Hsu et al. also from an area of HBV-associated HCC [24]. In this study, we have demonstrated that aberrant expression of cell surface Wnt co-receptor LRP6 may play an important role in the formation of HCC. To our knowledge, this is the first study to investigate the role of LRP6 and its expression pattern in HCC.

In general, four of our myc-CA LRP6 overexpressing clones showed more aggressive tumor phenotype in terms of the cell proliferation, migration, invasion and tumorigenicity assays. However, among these four stable clones, there was no definite relationship between their LRP6 expression levels and their β-catenin levels or aggressive phenotypes. Indeed, clone #3, with the weakest level of ectopic myc-CA LRP6 expression, showed the strongest aggressive phenotype as compared with the other clones with relatively higher protein expression levels of LRP6. This observation might probably be due to the clonal differences among those individual clones. Another explanation might be due to the deleterious effect of high amounts of LRP6 protein leading to the accumulation of LRP6 intracellular domain (LRP6-ICD) into the nucleus of stable cells. Indeed, LRP6-ICD was first reported to increase TCF/β-catenin activity and activate Wnt/β-catenin pathway [25]. However, further studies showed that it repressed TCF/β-catenin activity in CHO and HEK 293T cells when it translocated into the nucleus [26]. This LRP6-ICD was also found in all of our LRP6 stable clones (data not shown). Therefore, we speculate that ectopic expression of LRP6 in high levels may lead to the accumulation of excessive LRP6-ICD protein, which in turn may act as a negative feedback loop to control the Wnt/β-catenin signaling. Further investigation on the role of LRP6-ICD and its mechanism in HCC development is needed.

Cell surface Wnt co-receptor LRP6 was previously found to be essential for the activation of Wnt/β-catenin signaling upon binding with Wnt ligands [27], [28], [29]. Reports on the expression pattern and functional roles of LRP6 in cancers are scanty. Only very recently, Liu et al. showed that LRP6 was overexpressed in 20–36% of human breast cancers [13]. In this study, we showed that both transcript and protein levels of LRP6 were up-regulated in HCC. Upon ectopic expression of LRP6, the protein level of β-catenin was increased. Using TCF/β-catenin reporter assay, the β-catenin-dependent transcriptional activities were also found to be upregulated in these cell lines. Stable expression of constitutively active LRP6 in BEL-7402 HCC cells showed hyperactivation of Wnt/β-catenin pathway with higher level of β-catenin protein. The findings showed that upregulation of co-receptor LRP6 was able to increase the activities of Wnt/β-catenin signaling. In human HCCs, although 22% of cases showed upregulation of both LRP6 transcript and β-catenin protein levels by immunohistochemical staining, there was no significant correlation of LRP6 overexpression and β-catenin overexpression. It might be due to the fact that our sample size was small. Furthermore, overexpression of other Wnt elements, including Fizzled receptors and Dishevelled proteins, have also been reported to contribute to upregulate the β-catenin level [17], [30], [31]. Thus, it may be difficult to dissect the sole effect of LRP6 upon β-catenin in clinical samples.

Importantly, LRP6 enhanced cell proliferation, cell migration and invasion *in vitro*, as well as tumorigenicity *in vivo* in nude mice. Our findings support the role of LRP6 in hepatocarcinogenesis. In fact, similar findings have recently been reported in breast cancer model by Bu's group. They showed that either knockdown of LRP6 or LRP6 antagonist suppressed tumor growth [13]. Using MMTV-LRP6 mice model, they further confirmed that LRP6 activated Wnt signaling and contributed to breast cancer tumorigenesis *in vivo* [14]. By using different experimental approach, data from our present study and Bu's group have concordantly provided evidence that overexpression of cell-surface Wnt co-receptor LRP6 may play an important role in cancer progression through activation of the Wnt/β-catenin signaling pathway.

Evidence supporting the oncogenic role of those cell-surface Wnt receptors and co-receptors in carcinogenesis is accumulating. In addition to the aberrant expression of LRP6, overexpression of other cell surface Wnt receptors or co-receptors has also been found to play a significant role in the activation of Wnt/β-catenin pathway in HCC. Frizzled 7 receptor (FZD7) and other Frizzled receptors have been found to be overexpressed in human HCC and FZD7 upregulation was associated with activation of Wnt/β-catenin signaling [30], [31], [32]. Bengochea et al. assessed the gene expression profile of 10 Frizzled receptors in human HCC samples and showed that Frizzled-3/6/7 receptors were up-regulated in human HCC [33]. Frizzled receptors were also found to be overexpressed in other cancer types, like gastric and renal carcinoma and astrocytomas [34], [35], [36]. All these findings implicated the potential oncogenic role of the dysregulation of cell surface Wnt receptors.

In addition to LRP6, co-receptor LRP5 was also found to be implicated in Wnt/β-catenin pathway in cancers. In fact, both LRP5 and 6 have been suggested to be oncogenic proteins and could be a target for cancer therapy [37]. LRP5 was found to be overexpressed and correlated with tumor metastasis in high-grade osteosarcoma; blocking the LRP5 by its dominant negative form decreased tumorigenicity and metastasis of osteosarcoma [38], [39]. Furthermore, internally truncated form of LRP5 (LRP5Delta) has recently been reported to be resistant to DKK1 inhibition and thus contributes to the activation of Wnt/β-catenin signaling in parathyroid and breast cancer [40], [41]. These reports have revealed the role of LRP5 in oncogenesis. In our preliminary study, LRP5 was not significantly overexpressed in human HCCs and the LRP5Delta form was absent in human HCC cell lines (data not shown). Our findings suggest that LRP6 is more likely to have a pathogenic role than LRP5 in human HCC.

Overall, our findings suggest that overexpression of cell surface co-receptor LRP6 may contribute to the activation of Wnt/β-catenin signaling pathway in human HCCs and, in turn, play a role in hepatocarcinogenesis.

## Materials and Methods

### Ethics statement

Use of human tissue samples in this project was approved by the Institutional Review Board of the University of Hong Kong/Hospital Authority Hong Kong West Cluster. All patients gave his/her informed and written consent on the use of the clinical specimens for research. Animal work was preformed following the Animals (Control of Experiments) Ordinance (Hong Kong) and Institute's guidance on animal experiments. The protocol (CULATR 1132-05) was approved by the Committee on the Use of Live Animals in Teaching and Research of the University of Hong Kong.

### Patient samples and patients' demographic data

Sixty pairs of HCCs and their corresponding non-tumorous liver tissues were employed for real time-quantitative PCR (qPCR) analysis. Forty-three of the patients were male and 15 were female. The patients' ages ranged from 24 to 74 years (mean: 54±13 years). The clinicopathological features of the patients are summarized in Table 1. All patients had surgical resection at Queen Mary Hospital, The University of Hong Kong and were randomly selected for this study. The resected specimens were obtained immediately after surgical resection, snap-frozen in liquid nitrogen, and kept at −80°C. The non-tumorous liver samples were taken more than 1 cm away from the tumors.

### Plasmid constructs

The full-length (FL) myc-LRP6 pCS2+ and LRP6-EGFP pCS2+ were generous gifts from Dr. Christof Niehrs (Deutsches Krebsforschungszentrum, Heidelberg, Germany). Myc-tagged *LRP6* gene was then subcloned into pBABE-puro vector (myc-FL LRP6); the myc-FL LRP6 construct was digested with BspE1 and SexA1, end-filled and re-ligated to form the truncated myc-LRP6 without 4 EGF-like repeats. This truncated LRP6 is a constitutively-active form (myc-CA LRP6) [15]. Super8X Top-Flash and Super8X Fop-flash reporter constructs were kindly provided by Dr. Randall Moon (University of Washington, Seattle, WA).

### Cell culture

L cells, L-Wnt3A cells, Human embryonic kidney (HEK293T) cells, HCC cell lines PLC/PRF/5, HepG2 and Hep3B were purchased from the American Type Culture Collection (Manassas, VA). BEL-7402 and SMMC-7721 were obtained from the Shanghai Institute of Cell Biology. A pair of HCC primary and metastatic cell lines (H2P and H2M) was kindly provided by Dr. XY Guan [16]. BEL-7402, SMMC-7721 and HEK293T cells were maintained in DMEM with high glucose; HepG2, Hep3B and PLC/PRF/5 cells were maintained in MEM with 1 mM sodium pyruvate; H2P and H2M cells were maintained in DMEM with 1 mM sodium pyruvate. All media were supplemented with 10% fetal bovine serum (FBS), penicillin at 100 unit/ml and streptomycin at 100 µg/ml (Invitrogen, Gaithersburg, MD). All cells were incubated at 37°C in a humidified incubator containing 5% carbon dioxide.

### Preparation of Wnt3A conditioned medium

L or L-Wnt3A cells were grown on 10-cm plates and to 80–100% confluence. The cells were sub-cultured in a triple-layer flask filled with around 80 mL of medium. After 4 days, the culture medium was collected in Falcon tubes and centrifuged to remove cell debris. The medium was filtered through 0.2-µm membranes. Fresh medium was replenished and cells were allowed to grow for another 3 days, at which time a second batch of culture medium was collected in Falcon tubes.

### Establishment of stable clones

Myc-CA LRP6 stable clones were established to reveal the functional roles of LRP6 in BEL-7402 cell line. After transfection of myc-CA LRP6 and vector pBABE-puro, cells were maintained in 5 µg/ml puromycin and allowed to grow until distinct colonies were distinguished, at around 4 weeks' time. Single clones were isolated and expanded in medium supplemented with 1 µg/ml puromycin. Each clone was then checked for the overexpression of the myc-CA LRP6 by Western blotting.

### Immunohistochemical staining

Immunohistochemical staining for LRP6 was performed on formalin-fixed, paraffin-embedded sections using the labeled streptavidin-biotin method. For antigen retrieval, sections were immersed in buffer with 1 mM EDTA and boiled for 15 min. Polyclonal rabbit anti-human LRP6 antibody (Cell signaling, Danvers, MA) was used at 1∶100 dilution. For negative control, the primary antibody was replaced with Tris-buffered saline.

### Western blotting

Total protein from cultured cells was extracted in RIPA lysis buffer (50 mM, pH 7.4 Tris-HCl, 1% NP-40, 0.25% Na-deoxycholate, 150 mM NaCl, 1 mM EDTA, 5 mmol/L sodium fluoride) with freshly added Complete EDTA-free Protease Inhibitor Cocktail Tablets (Roche, Mannheim, Germany). Twenty µg samples were loaded in each lane for SDS-PAGE and then transferred to polyvinylidene difluoride membrane for immunoblotting. Blots were blocked and followed by incubation with antibodies specific for anti-LRP6 (#2560; Cell signaling), anti-β-catenin (#610153; BD transduction lab, San Jose, CA), anti-c-Myc (9E10) (sc-40; Santa Cruz, Santa Cruz, CA) and anti-β-actin (A5316; Sigma, MO, USA). Blots were then incubated with anti-mouse or anti-rabbit HRP antibody at 1∶5000 dilution (GE Healthcare Life Sciences, Piscataway, NJ).

### Luciferase assay

β-catenin activity was measured by luciferase reporter assay of TCF/LEF-dependent transcription (TOP/FOP reporter assay). HCC cells or HEK293 cells were seeded in 24-well plates. Then, either vector or LRP6 construct was co-transfected with pSuper8XTOPflash or pSuper8XFOPflash luciferase reporter construct. Renilla luciferase construct pRL-CMV was also co-transfected into each well for normalization. The luciferase activity was measured using the Dual-Luciferase Reporter assay system (Promega) as described previously [17].

### RT-PCR analysis

Total RNA was extracted from HCC cell lines and human HCC samples by TRIzol reagent (Invitrogen). First-strand cDNA was synthesized from 1 µg of total RNA using random hexamers with GeneAmp RNA PCR Kit (Applied Biosystems, Foster City, CA). The cDNA was then used for detecting the expression level of LRP6 (Hs00233945_m1) with TaqMan Gene Expression Assays (Applied Biosystems). Quantitative PCR (qPCR) was performed in Applied Biosystems 7900HT Fast Real-Time System (Applied Biosystems) in triplicates. The expression of target genes in paired HCC and non-tumorous liver tissues from the same patient was expressed as T/NT ratio. A T/NT ratio >2 was defined as overexpression, whereas a T/NT ratio <0.5 was defined as underexpression.

### *In vitro* proliferation assay

Cell proliferation of LRP6 expressing cells was determined using cell counter. In brief, 5×10^4^ of cells were seeded onto 6-well plates in triplicates for each counting day for 7-day proliferation assay. Cells were counted on consecutive days for a week and proliferation curves were plotted.

### *In vitro* cell migration and invasion assays

Cell migration assay was performed using transwell inserts with polycarbonate membranes of 8.0-µm pore size (Corning Inc., NY). Matrigel invasion assay was done using transwell insert pre-coated with Matrigel (BD Biosciences, San Jose, CA). In brief, 1×10^5^ cells in serum-free medium were seeded onto the upper chamber. Culture medium containing 10% FBS was used as a chemoattractant in the lower chamber. Cells were incubated in a humidified incubator at 37°C for 18 h (for migration assay) or 20 h (for invasion assay). Migrated or invaded cells on the lower surface of the membrane were then fixed with methanol, stained with crystal violet, photographed and counted in 5 random fields. Each experiment was performed thrice.

### *In vivo* tumorigenicity assay

To assess the tumorigenicity, cells were trypsinized and resuspended in phosphate-buffered saline. Cells (2×10^6^) were injected subcutaneously into the flank of 6-week-old male BALB/c nude mice using a 25-gauge needle (n = 5 for each group of experiments). Tumor size was monitored weekly by measuring the largest and smallest diameters of tumor and estimated according to the formula: volume = 1/2×(largest diameter)×(smallest diameter) ^2^.

### Statistical analysis

The clinicopathologic features of HCC patients were analyzed by SPSS for Windows 17.0 (SPSS Inc., Chicago, IL). Data from real-time qPCR were analyzed using Prism 5 software (GraphPad Software Inc., CA, USA). Mann-Whitney test was used for analysis between tumor and non-tumor groups, and Student's t-test for comparing cell numbers in cell proliferation, migration and invasion assays. Tests were considered significant if their P values were less than 0.05.

## Supporting Information

Figure S1 **Constitutively active LRP6 promoted both cell migration and invasion.** Cell migration and invasion assays were performed using LRP6-stably expressing BEL-7402 cells. (A) Overexpression of myc-CA LRP6 enhanced cell migration in BEL-7402 cells. The numbers of migrated cells in CA LRP6 Clones #1 and #11 (P<0.001 and  = 0.016, respectively) were significantly higher than the vector control. (B) Overexpression of myc-CA LRP6 promoted cell invasion in BEL-7402 cells. The numbers of invaded cells in CA-LRP6 Clones #1 and #11 were significantly higher than the vector control cells (P<0.019 and  = 0.016, respectively).
(TIF)

Figure S2 **Constitutively active LRP6 enhanced tumor cell growth** ***in vivo*** **.** (A) *In vivo* nude mice injection assay was performed by injecting myc-CA LRP6 stably expressing and vector control BEL-7402 cells subcutaneously into the flank of the nude mice. (B) Tumor sizes of two myc-CA LRP6 stably expressing tumors, Clones #1 and #11, were significantly higher as compared with the tumor of vector control (P = 0.003 and 0.001, respectively). (C) The tumor weights of Clones #1 and #11 showed a trend of higher tumor weight although the difference did not reach statistical significance (P = 0.093 and 0.111, respectively). Error bar = SEM.
(TIF)
