# Supplementary figures and images for: Upregulation of the Wnt Co-Receptor LRP6 Promotes Hepatocarcinogenesis and Enhances Cell Invasion
Source: PLoS One. 2012 May 3;7(5):e36565. doi: 10.1371/journal.pone.0036565 (PMC3343020; doi:10.1371/journal.pone.0036565)

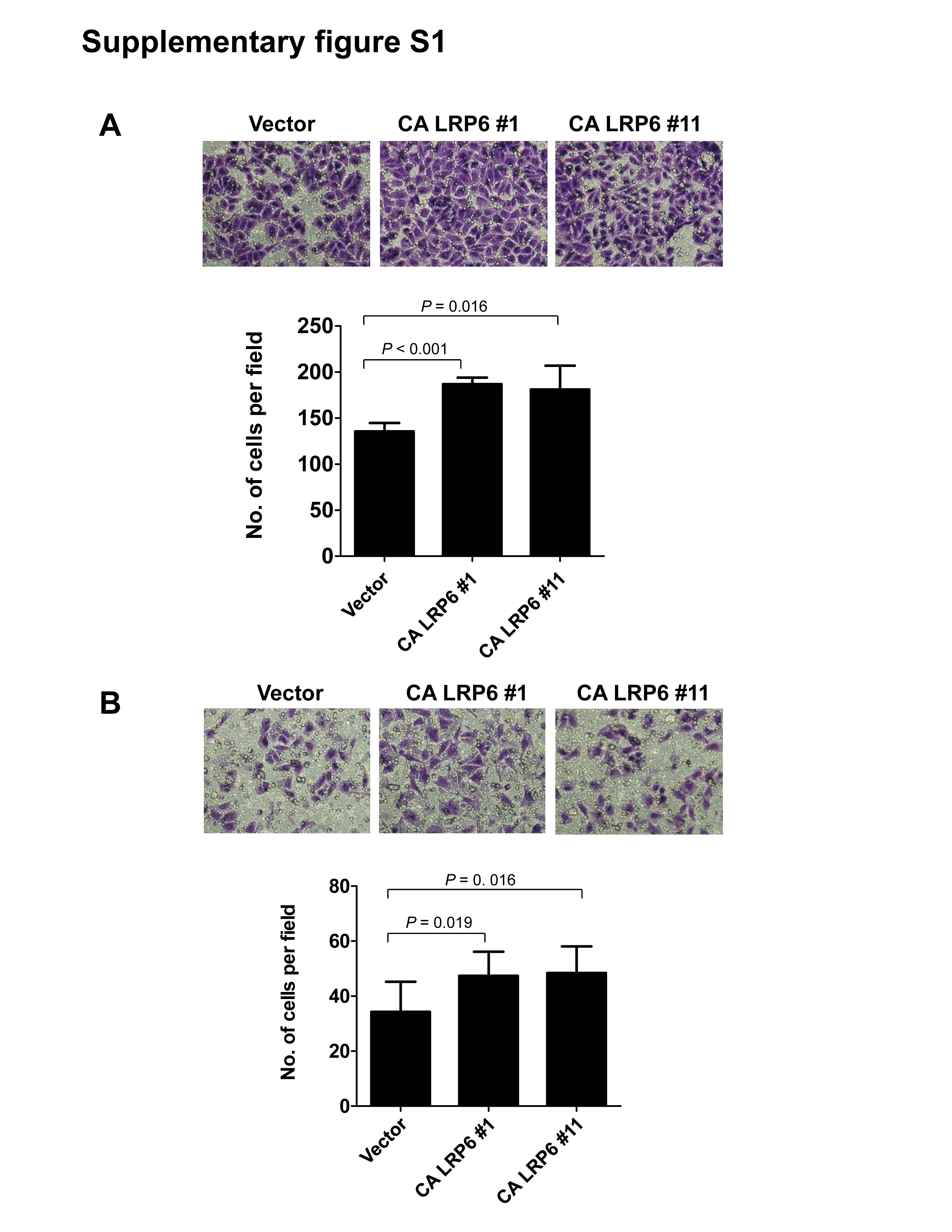

Supplement: Figure S1 — Constitutively active LRP6 promoted both cell migration and invasion. Cell migration and invasion assays were performed using LRP6-stably expressing BEL-7402 cells. (A) Overexpression of myc-CA LRP6 enhanced cell migration in BEL-7402 cells. The numbers of migrated cells in CA LRP6 Clones #1 and #11 (P<0.001 and = 0.016, respectively) were significantly higher than the vector control. (B) Overexpression of myc-CA LRP6 promoted cell invasion in BEL-7402 cells. The numbers of invaded cells in CA-LRP6 Clones #1 and #11 were significantly higher than the vector control cells (P<0.019 and = 0.016, respectively). (TIF) [file pone.0036565.s001.tif]

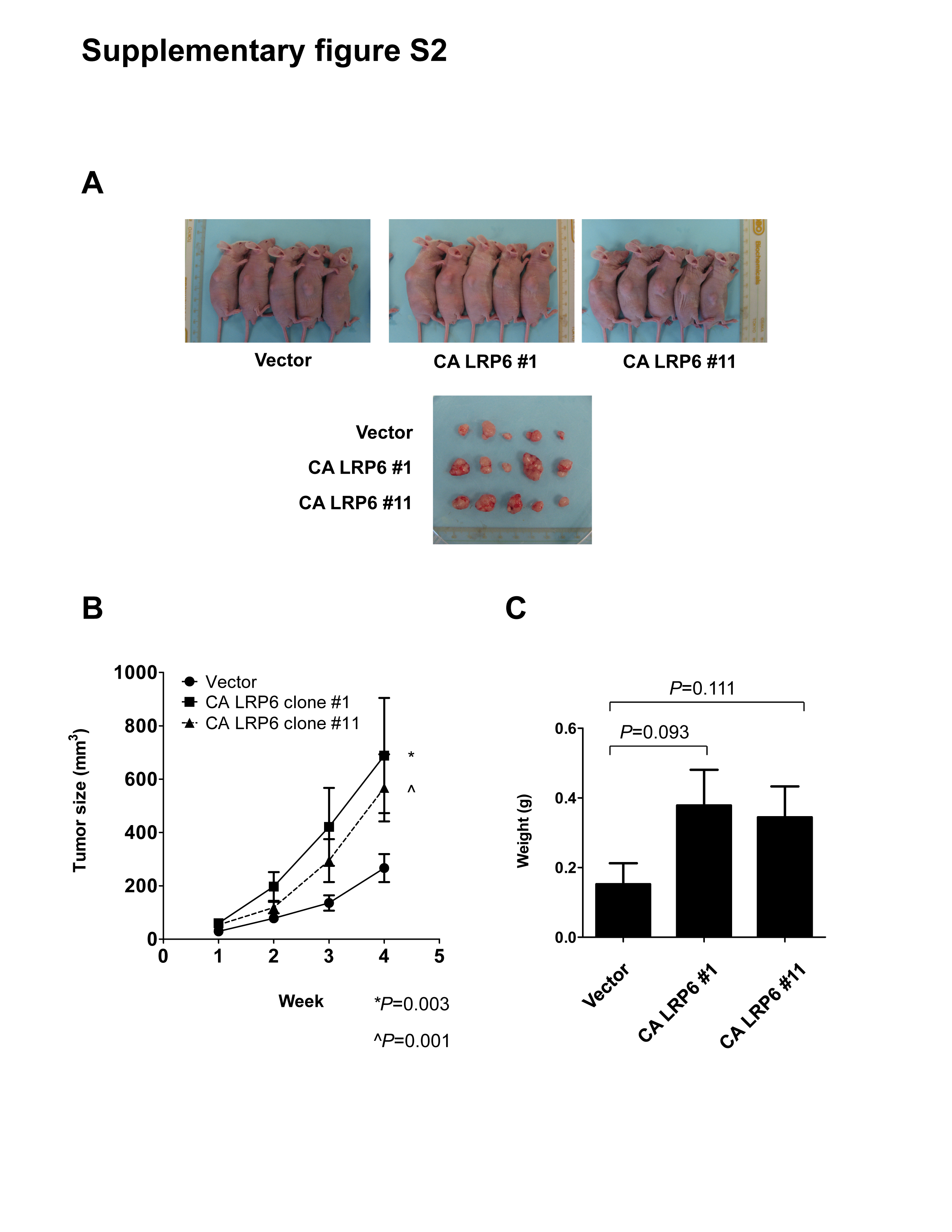

Supplement: Figure S2 — Constitutively active LRP6 enhanced tumor cell growth in vivo . (A) In vivo nude mice injection assay was performed by injecting myc-CA LRP6 stably expressing and vector control BEL-7402 cells subcutaneously into the flank of the nude mice. (B) Tumor sizes of two myc-CA LRP6 stably expressing tumors, Clones #1 and #11, were significantly higher as compared with the tumor of vector control (P = 0.003 and 0.001, respectively). (C) The tumor weights of Clones #1 and #11 showed a trend of higher tumor weight although the difference did not reach statistical significance (P = 0.093 and 0.111, respectively). Error bar = SEM. (TIF) [file pone.0036565.s002.tif]
